# Supplementary material for: Morphology Control and Metallization of Porous Polymers Synthesized by Michael Addition Reactions of a Multi-Functional Acrylamide with a Diamine
Source: Materials (Basel). 2021 Feb 9;14(4):800. doi: 10.3390/ma14040800 (PMC7915525; doi:10.3390/ma14040800)
Supplement: Supplementary file 1 [file materials-14-00800-s001.pdf]

Supplementary Material

Morphology Control and Metallization of Porous Polymers Synthesized by Michael Addition Reactions of a Multi-Functional Acrylamide with a Diamine

Naofumi Naga [1,2,*], Minako Ito [2], Aya Mezaki [1], Hao-Chun Tang [3], Tso-Fu Mark Chang [3], Masato Sone [3], Hassan Nageh [4] and Tamaki Nakano [4,5]

[1] Department of Applied Chemistry, College of Engineering, Shibaura Institute of Technology, 3-7-5 Toyosu, Koto-ku, Tokyo 135-8548, Japan; ad14094@shibaura-it.ac.jp
[2] Graduate School of Science & Engineering, Shibaura Institute of Technology, 3-7-5 Toyosu, Koto-ku, Tokyo 135-8548, Japan; mc19002@shibaura-it.ac.jp
[3] Institute of Innovative Research, Tokyo Institute of Technology, Yokohama, Kanagawa 226-8503, Japan; hct2498@gmail.com (H.-C.T.); chang.m.aa@m.titech.ac.jp (T.-F.M.C.); sone.m.aa@m.titech.ac.jp (M.S.)
[4] Institute for Catalysis and Graduate School of Chemical Sciences and Engineering, Hokkaido University, N 21, W 10, Kita-ku Sapporo 001-0021, Japan; science_as2000@yahoo.com (H.N.); tamaki.nakano@cat.hokudai.ac.jp (T.N.)
[5] Integrated Research Consortium on Chemical Sciences, Institute for Catalysis, Hokkaido University, N 21, W 10, Kita-ku Sapporo 001-0021, Japan
* Correspondence: nnaga@sic.shibaura-it.ac.jp

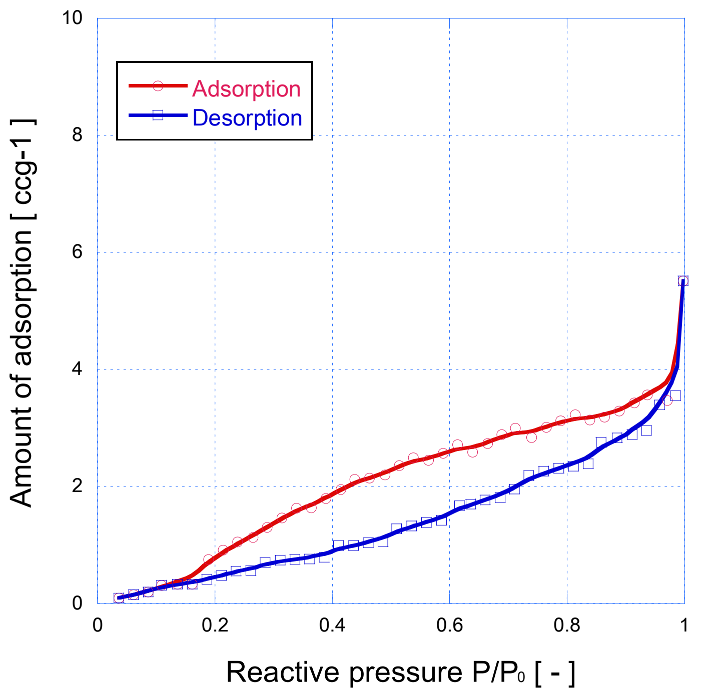

histogram

Figure S1. Nitrogen adsorption-desorption isotherms of AM4/HDA porous polymer I-10w-25d.

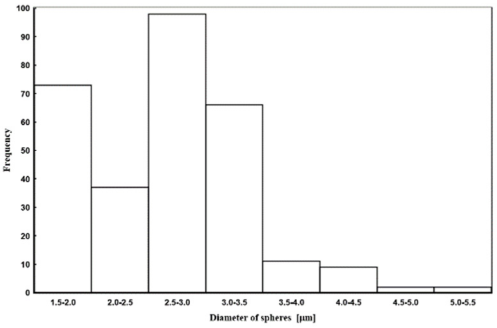

(a)

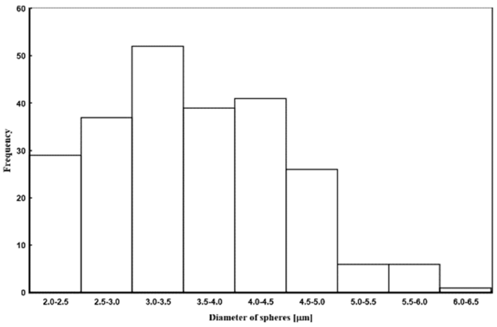

(b)

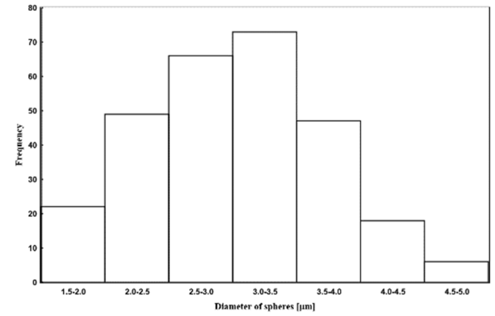

(c)

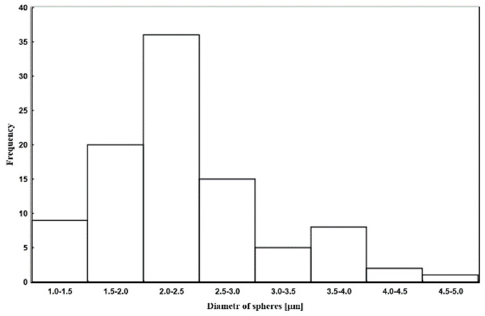

(d)-i

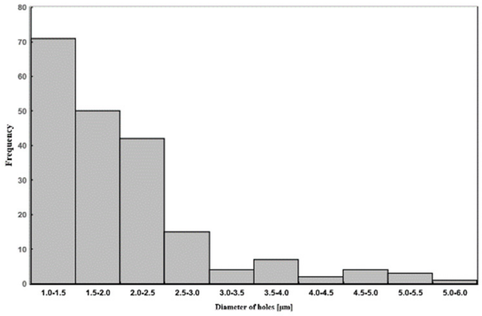

(d)-ii

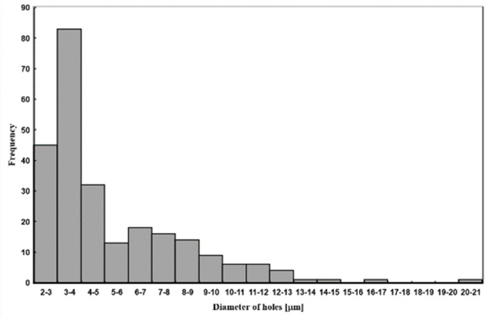

(e)

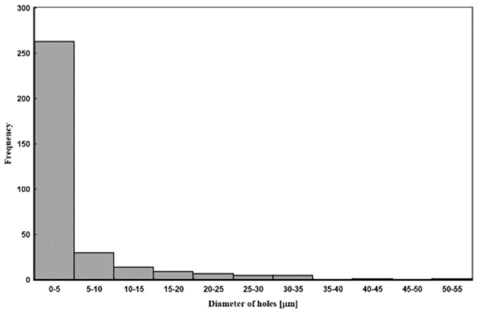

(f)

Figure S2. Size distribution of spheres and/or holes of AM4-HDA porous polymers Case I (feed molar ratio of AM4/HDA: 1/2), monomer concentration and temperature (a) I-20w-70d (spheres), (b) I-20w-80d (spheres), (c) I-20w-90d (spheres), (d) I-25w-80d (i: spheres, ii: holes), (e) I-25w-90d (holes), and (f) I-30w-90d (holes).

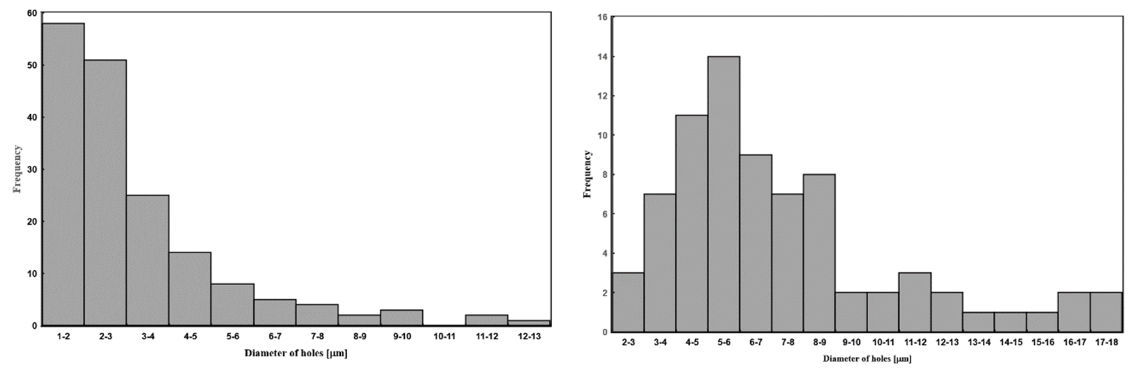

(b) (c)

Figure S3. Size distribution of holes of AM4-HDA porous polymers Case II (feed molar ratio of AM4/HDA: 1/1), concentration and temperature (b) II-25w-60d and (c) II-25w-80d.

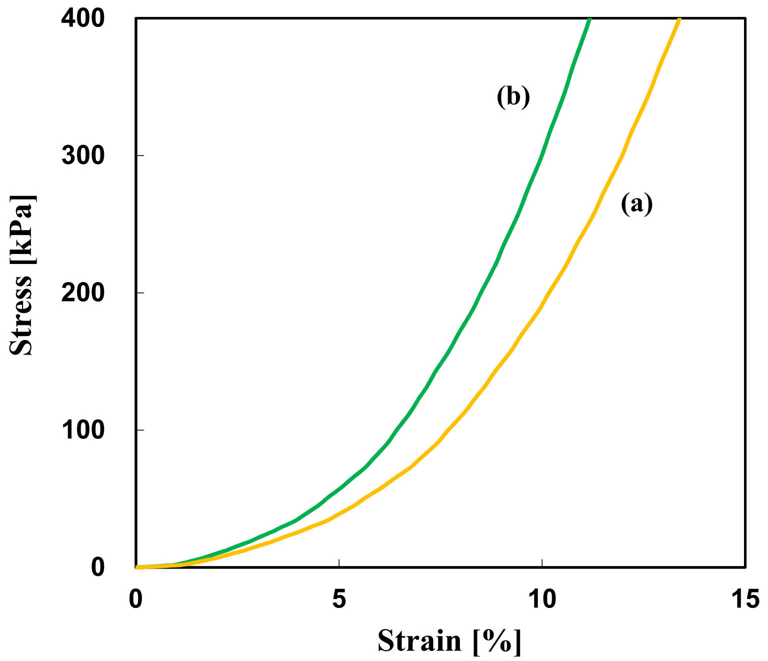

Figure S4. Stress-strain curves of AM4-HDA porous polymers (a) I-30w-90d and (b) II-30w-90d.

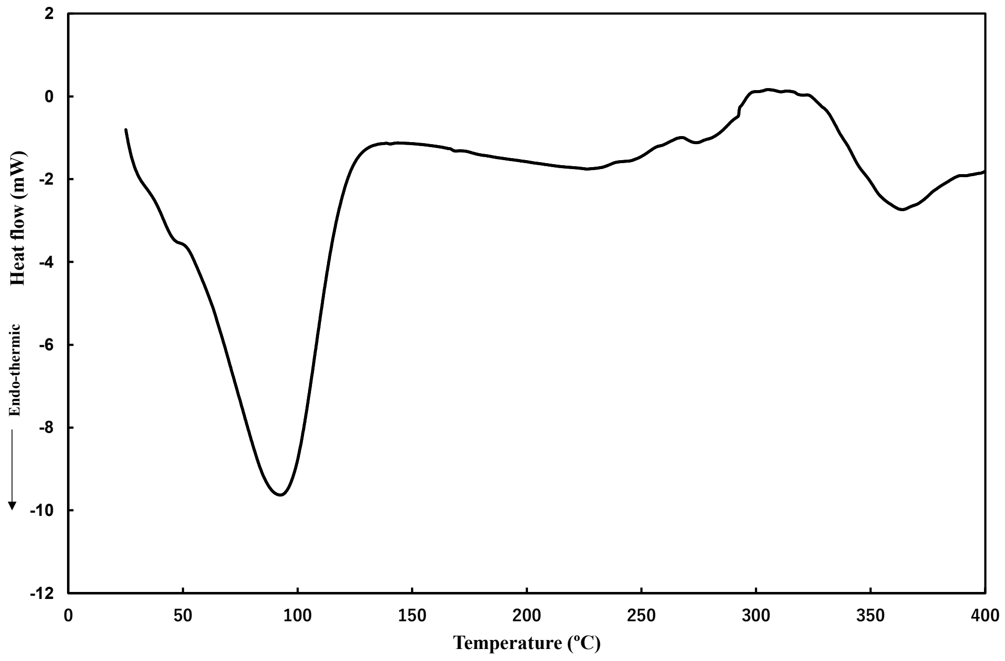

Figure S5. DSC profile of AM4-HDA porous polymer I-10w-25d.

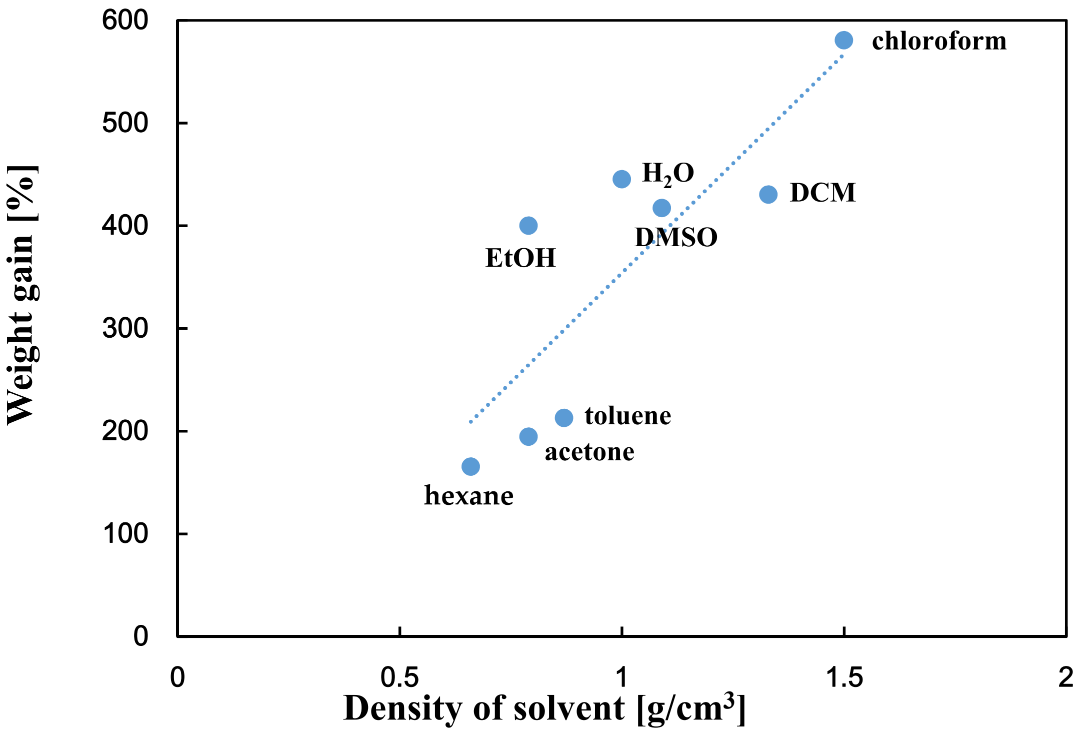

Figure S6. Correlation between absorption capacity of AM4-HDA porous polymer I-10w-25d and density of solvent.

Absorption capacity of AM4-HDA porous polymer.

Solvent	Density of solvent [g/cm^3]	Weight gain [%]
chloroform	1.50	580.6
DCM	1.33	430.3
toluene	0.87	212.7
hexane	0.66	165.4
EtOH	0.79	400.0
acetone	0.79	194.5
H_2O	1.00	445.3
DMSO	1.09	417.2

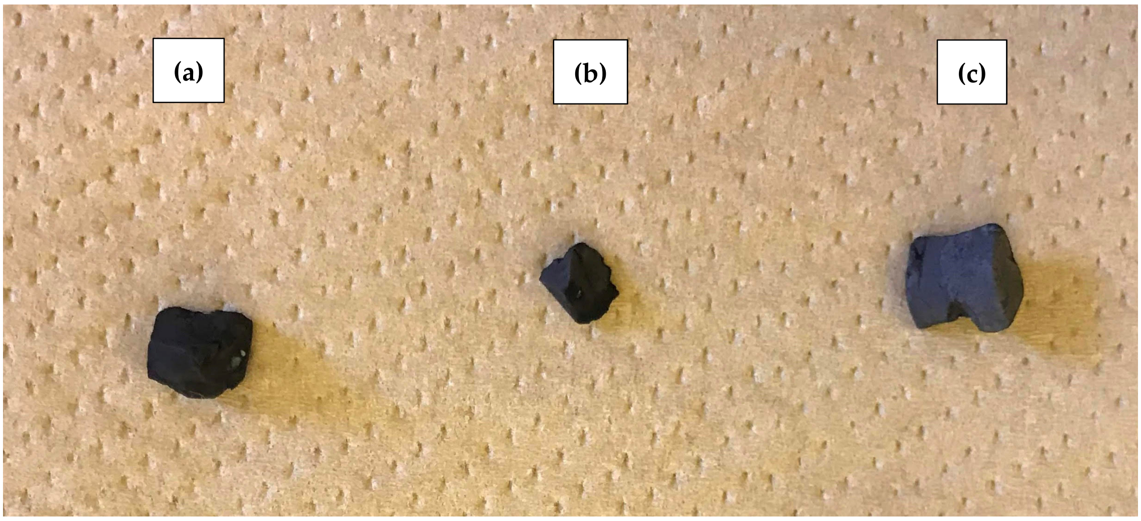

Figure S7. Photo images of the Ni plated AM-HDA porous polymer I-10w-60d, plating time: (a) 5 min, (b) 10 min, and (c) 20 min.
